# Lived experiences of adults’ non-compliance with psychiatric medication for depression

**DOI:** 10.4102/sajpsychiatry.v30i0.2202

**Published:** 2024-09-20

**Authors:** Jeanne du Plessis, Annie Temane, Marie Poggenpoel

**Affiliations:** 1 Department of Nursing, Faculty of Health Sciences, University of Johannesburg, Doornfontein, South Africa

**Keywords:** adults, depression, experiences, mental health, non-compliance, nursing, qualitative research

## Abstract

**Background:**

Non-compliance with psychiatric medication among patients diagnosed with depression ranges from 28% to 52% exacerbating illness and reducing treatment effectiveness. There is a paucity of research on medication non-compliance and its causes in South Africa and globally, and an urgent need to develop appropriate interventions.

**Aim:**

This study aimed to explore and describe the experiences of adults living with depression who are non-compliant with their psychiatric medication and formulate recommendations to facilitate their medication compliance.

**Setting:**

The study was conducted in a psychiatric ward at a public hospital in Gauteng, South Africa.

**Methods:**

The study employed a qualitative, exploratory, descriptive and contextual research design. Ten adults’ lived experiences were explored using in-depth individual interviews, and Tech’s coding method was used to analyse data.

**Results:**

Two themes emerged from the data: adults living with major depression offered several reasons for non-compliance, and adults living with major depression experienced non-compliance, which created a setback to their recovery.

**Conclusion:**

Non-compliance with medication is a common challenge among adults receiving mental health care and treatment. Ensuring compliance to medication is crucial for improving the prognosis of psychiatric conditions. Therefore, it is essential for healthcare practitioners in the field of psychiatry to have a comprehensive understanding of medication compliance and to effectively address any challenges that may arise in this area.

**Contribution:**

This paper contributes to the research field and adds knowledge to clinical nursing practice by exploring adults’ experiences with non-compliance to psychiatric medications while living with depression in the South African context.

## Introduction

Depression is a common mental health condition prevalent in most communities worldwide. It can have a negative impact on normal functioning, cause negative thoughts and significantly impact the afflicted individual’s quality of life.^1,2^ Depression has a global prevalence of 16.0% and was estimated to be the second-most prevalent illness in 2020, with an increase of 6.2% expected by 2030.^3^ In South Africa, major depressive disorder was estimated at 9.8% and depressive symptoms were reported at 26.0% in a 2014 South African social attitudes survey of the general population.^4,5^ Non-compliance with psychiatric medication among patients diagnosed with depression is 28% – 52%, with 40% of patients stopping their medication within a year and 75% by the end of 2 years.^6^

These statistics are concerning since living with depression can be a challenging and isolating journey, leading many individuals to rely on psychiatric medications to alleviate their symptoms.^7^ While these medications may prove beneficial for some, it is crucial to acknowledge the significance of adults’ personal experiences with non-compliance in taking psychiatric medications. The decision to embark on this treatment path can be intricate for many adults living with depression; uncertainty and discomfort regarding psychiatric medication’s long-term consequences are standard.^8^ Adults often find themselves torn between whether or not to embrace medication as part of their treatment plan.^9^

Adults living with depression face various challenges in complying with psychiatric medication. Moreover, the consequences of non-compliance with medication for the individuals’ family members and the healthcare system are of concern.^10^ Conversely, addressing and effectively treating fears of psychiatric medication non-compliance can improve individuals’ mental health, reduce the burden of relapse and lower the cost of mental health services.^11^ Considering the complex problems that non-compliance may exacerbate, it is important to assess its impact on adults living with depression and their experiences.

As a psychiatric ward worker in Gauteng, the researcher observed frequent readmissions of adults with depression because of their non-compliance with psychiatric medication. This prompted the researcher to pursue these individuals so that they could share their challenges with psychiatric medication compliance. Medication non-compliance can lead to long-term psychological problems, such as exacerbating symptoms or prompting relapse.^12^ Moreover, non-compliance of medication can have detrimental implications on the quality of life for psychological health, physical health and social relationships.^13^ Antidepressants are the most important treatment approach for depression, and non-compliance can thus be detrimental to individuals.^14^ Given the scope of the problem presented by depression, a wealth of research has been conducted on the causes, consequences and treatments for depression. However, there is a paucity of research on patients’ experiences, acceptance and medication compliance.^15^

Limited studies have explored medication non-compliance and its causes in South Africa and globally. There is therefore, a need to develop practically appropriate interventions and improve understanding of this phenomenon. To improve individuals’ psychiatric medication compliance, opportunities are needed so that patients can share their lived experiences with others, increase society’s awareness of the condition, and relieve them from their burden. This article aims to explore and describe the experiences of adults living with depression who are non-compliant with their psychiatric medication. Recommendations to facilitate compliance with their prescribed psychiatric medication will also be formulated.

## Research methods and design

A qualitative, exploratory, descriptive and contextual research design was employed to describe individuals living with depression’s experiences of non-compliance with psychiatric medication.^16,17,18^

### Setting

The research was conducted in a public hospital north of Gauteng, South Africa. Gauteng is one of the smallest provinces in South Africa, covering approximately 18 178 km.^19,20^ The hospital has one psychiatric ward, with a total bed capacity of 18, comprising 9 beds for males, 7 for females, and 2 uni-gender beds. This psychiatric ward admits individuals with various mental healthcare needs. The interviews were conducted in a dedicated psychologist’s room to ensure utmost privacy. The interviews lasted 45–60 min each (this has been mentioned under data collection also).

### Research population and sampling strategy

The population for this study was adults admitted to the psychiatric ward of a public hospital. The purposive sampling approach was used to gather data.^21^ A total of 10 individuals living with depression who were not complying with their psychiatric medication thus took part in the interviews until data saturation was reached. The inclusion criteria were adults living with depression who were admitted to a hospital’s psychiatric ward, were willing to participate and were non-compliant with medication. Adults who were male or female between 25 and 55 years were included, and participants had to be able to communicate in English. The sample size was determined during data collection when saturation was reached.^17^

### Data collection

Different data collection methods were applied in this study. In-depth phenomenological individual interviews, observations and field notes were all part of the process. A total of 10 participants were interviewed, and the researcher began the interview by asking a central question: ‘What is it like not to take your medication as prescribed by your doctor?’ The researcher’s main goal was to determine how the participants responded to the open-ended questions. The interviews lasted 45–60 min each and were audio recorded. The adults living with depression who were non-compliant with their psychiatric medications addressed the central question, and the researcher probed for further information until the entire experience was described. The researcher used the following communication tools during the interviews: clarification, reflection, probing and summarising.

### Data analysis

Tesch’s thematic coding method^22^ was used to analyse the audio-recorded interviews, observations and field notes. Data collection and analysis occurred concurrently. The audio recordings were listened to and transcribed, and field notes were read to make sense of the whole. Themes and categories were identified, and an independent coder who is an expert in qualitative research analysed the data separately, and a consensus discussion was held with the researcher about the findings. The results were structured around identified themes, supported by direct quotations from the data and substantiated by relevant literature.

### Rigour

The following criteria of trustworthiness were applied in the study: credibility, transferability, dependability and confirmability.^23^ Credibility was promoted through prolonged engagement; the researcher spent 2 months conducting the in-depth interviews. Data were collected from participants by an independent field worker to avoid coercion and any bias that the researcher could bring into the data collection process. In addition, data were independently analysed by the researcher and an independent coder. The researcher used multiple data collection methods, including interviews, observations and field notes to ensure data triangulation.^24^ To promote transferability, the researcher provided a thick description of the participants’ demographics and a rich description of the findings, with direct quotations from participants. A dense description was also provided of the study’s recommendations.^24^

To promote dependability, the researcher collected data until saturation was achieved. A dense description of the research process was also given, and the study’s findings were supported by appropriate data.^24^ The study’s dependability was also enhanced by the researcher carefully documenting all the research activities, conclusions and changes that occurred as the research evolved. For the confirmability criterion to be achieved, the researcher ensured that the findings reflected the participants’ voices and conditions of the inquiry, not the researcher’s biases, motivations or perspectives. A chain of evidence of the research process was also maintained.^24^

### Ethical considerations

Permission to conduct the study was acquired from the University of Johannesburg’s Research Ethics Committee (No. REC-673-2020), the University of Pretoria’s Ethics Committee (No. 737/2020), and the National Health Research Database (No. GP 202102_021). Furthermore, the concepts of autonomy, beneficence, non-maleficence and justice were used.^17^ Participants received an explanation and information letter about the proposed study, which included information about their right to voluntary participation, the right to withdraw without penalty, a request to sign the informed consent form after reading and understanding the letter, a request to record the interview, and assurances of confidentiality, anonymity, and privacy. Although there were no direct advantages to participating in the study, recommendations were developed and distributed to help care for individuals with depression who are non-compliant with their psychiatric medication. Moreover, the researcher ensured the participants were not harmed during the interviews and were recruited fairly during the selection procedure. In case of emotional discomfort during the interview, the independent field worker facilitated debriefing by referring the participant to a counsellor and asking questions or airing complaints. The recorded interviews and transcripts were stored in a password-encrypted computer file in the researcher’s laptop. The data will be kept for 10 years after the study’s publication, and will only be accessible to the researcher and supervisors.

## Results

The results and discussion section are presented as an integrated section next. The study included 10 adult participants (5 male and 5 female) aged 25 to 55 years who were not complying with their psychiatric medication as recommended. Of the 10 participants, 5 were black and 5 were white; the ethnicity is to capture a wide range of views and experiences. The participants had been living with depression between 2–10 years. The participants’ characteristics are represented in Table 1.

**TABLE 1:** Participants’ characteristics.

Participant	Gender	Age (years)	Employment status	Ethnicity	Years living with depression
P1	Male	26	Unemployed	Black	2
P3	Male	42	Employed	Black	6
P7	Male	48	Employed	White	5
P9	Male	34	Employed	Black	3
P10	Male	25	Unemployed	Black	2
P2	Female	42	Employed	White	4
P4	Female	48	Employed	White	8
P5	Female	55	Unemployed	White	10
P6	Female	36	Employed	Black	3
P8	Female	39	Employed	White	7

*Source*: Adapted from du Plessis^27^

Two themes emerged from the analysed data:

1. Adults living with major depression offered several reasons for non-compliance.
2. Adults living with major depression experienced non-compliance, which created a setback to their recovery. Table 2 summarises the themes that emerged from the collected data.

**TABLE 2:** Themes of adults’ lived experiences of non-compliance with psychiatric medications while living with depression.

Themes	Categories
**Theme 1:** Adults living with depression are non-compliant with medication for various reasons	**Category 1:** Expectations of medication were unmet for the following reasons: The medication was ineffective Challenges in effectively managing the side effects associated with depression medication have emerged as a significant concern Mental illness relapse
	**Category 2:** Adults living with depression described interpersonal challenges as reasons for non-compliance with medication. These challenges included: Poor personal relationships Stigma
	**Category 3:** Social circumstances impacted adults living with depression’s compliance with medication, including: A lack of support Financial constraints
**Theme 2:** Adults living with major depression experienced non-compliance, which created a setback to their recovery	**Category 1:** Perceptions of medication changed when adults living with depression realised non-compliance impacted their lives. They consequently: Experienced support from friends and family Experienced concern over their mental health

*Source:* Adapted from du Plessis^27^

## Theme 1: Adults living with major depression offered several reasons for non-compliance

Adults living with depression experienced that the expectations of medication were unmet; they described interpersonal challenges as reasons for non-compliance with medication and lastly, they experienced that social circumstances impacted their compliance with medication. These are described below.

### Category 1: Expectations of medication were unmet

A majority of adults living with depression expressed that their expectations of their antidepressant medication were unmet. The participants further shared their understanding about the mental health condition they were diagnosed with, and the effects of the medication were limited. The sub-categories discussed next emerged from the study:

#### The medication was ineffective

The study’s findings revealed that the adult participants’ experiences with the condition and the effects of the medicine were minimal at the outset of treatment, and they tended to use a subjective rather than an objective set of notions when evaluating the effects of the medication. A participant made the following statement:

‘It is not easy. Somehow you feel the medication is not working, and then you no longer want to take the medication.’ (Participant 3, 42-year-old, Male)

According to Jackson, Chamberlin and Kroenke,^25^ when patients see an improvement in their symptoms over time, their satisfaction with therapy rises and vice versa. The patient’s illness representation and beliefs about causality, control or cure and consequences influence the information they require from their health providers,^26^ as well as their desire to engage in and comply with treatment.^19^

In this study, adult participants did not view their medication as the only treatment modality that was considered, but rather saw them as one component that could help them improve their mental health and daily activities. Although many adult participants did not take their doctors’ prescribed medications, others tried them in various ways. A variation in the use of medicine by adult participants was observed, especially if the patient’s medication was changed. They often felt more comfortable to use their ‘old medication’ especially when they experienced side-effects from the new medication. This attests to their experiences of medication not working, as the following quote suggests:

‘And I do not like the type of medication. I was much better on the old medicine. I will not take it. It does not make me feel good at all. I panic. I am paranoid. I do not want to wet my bed. I felt much better with the medication I was on.’ (Participant 5, 55-year-old, Female)

Non-compliance of psychiatric medication is a complex issue that encompasses patient-related and environmental factors. For example, patient factors include worries about side effects, medication costs, fear of addiction, and environmental factors include cultural and attitudinal problems, and clinician-related factors such as a lack of adequate patient education and shared decision-making, as well as poor follow-up.^27^ Evidence suggests that the mode in which psychiatric medication is originally given predicts patient treatment compliance and outcome.^28^ Compliance with psychiatric medication is critical for conducive treatment results while treating depressive disorders. Poor compliance rates are thus especially alarming from a therapeutic standpoint because compliance to psychiatric medication is essential for effective treatment outcomes.

#### Challenges in effectively managing the side effects associated with depression medication have emerged as a significant concern

The study’s findings suggested that antidepressant medication is linked with various side effects, which are difficult to treat and can result in secondary negative and cognitive symptoms. As reported by most participants, such side effects contribute to non-compliance with treatment and worsen results. Moreover, balancing the advantages of pharmaceutical treatments against their side effects is difficult since side effects can severely affect the participants’ quality of life. Regardless of medication side effects, mental illnesses decrease cognitive functioning and alter insight, complicating treatment decision-making, as experienced by all the participants.^29^ For these reasons, deciding on pharmacotherapy as a treatment modality is complex and challenging for patients, caregivers and physicians. A participant claimed:

‘The thing is, and then it, it makes my feelings ‘bietjie’ (a little) mixed up, because then sometimes I am happy, sometimes I am sad. And then sometimes I am just there.’ (Participant 2, 42-year-old, Female)

A recent study shows that antidepressant medication has been recognised to cause withdrawal symptoms in a substantial proportion of users.^30^ While such reactions may be moderate, short-lived, and controllable with reassurance and explanation for some people,^31^ in others, even with gradual withdrawal, these reactions are strong and long-lasting. They can make regular functioning impossible.^32^ Thus, antidepressant medications might induce withdrawal symptoms if the patient stops taking them, especially if they have been on these medications for a long time.^33^

#### Mental illness relapse

Adults with depression who do not take their psychiatric medication may experience psychiatric medication relapses. Participants reported that their experiences made them feel weary, stressed and burdened, which harmed their mental and physical health. The adults found it challenging to cope with the relapses they were experiencing. The following quote supports this view:

‘No, I relapse and have the problem of locking up and being admitted to a hospital. I end up losing weight and come to release I do not take my medication as I must after the doctors explain to me and understand for me not to take medication, I am going to lose my life.’ (Participant 3, 42-year-old, Male)

On a global basis, depression is the leading cause of disability.^1^ Even though short-term therapies for acute periods of severe depression are typically successful, many patients experience relapses with early recurrence of symptoms within the predicted period of a current episode, perhaps 3–12 months, or later recurrences of new episodes following initial short-term recovery or remission.^34^ Because health institutions have not yet fully responded to the burden of mental disorders, there is a massive gap between the demand for treatment and its availability worldwide.^35^ As a result, between 76% and 85% of patients with mental illnesses in low and middle-income countries do not receive treatment, resulting in relapse.^36^

### Category 2: Adults living with depression described interpersonal challenges as reasons for non-compliance with medication

The participants in this study who faced intrapersonal and interpersonal challenges with medication compliance reiterated their concerns about how others saw them. These challenges are discussed next.

#### Poor personal relationships

Personal relationships affected the behaviour of adults living with depression and their compliance with medication. They consequently experienced poor personal relationships with others. A participant shared:

‘They just get angry and violent towards me. That just makes you say I do not want anything anymore … I would take that medication. If the circumstances were not there.’ (Participant 8, 39-year-old, Female)

Another participant shared:

‘[*H*]ave no shoulder or pillar to cry on, and my that makes me not take my medication.’ (Participant 3, 42-year-old, Male)

Depressive patients are particularly susceptible to experiencing challenges in their interpersonal relationships because of the symptoms associated with the illness. Research has revealed various connections between interpersonal relationships, depressive symptoms and perceived social support. A study conducted by Berzonsky and Kinney^37^ discovered that individuals with heightened depressive symptoms expressed greater difficulty in engaging with others. These difficulties were attributed to negative self-evaluations and feelings of anxiety. Individuals exhibiting higher levels of depressive symptoms thus reported significantly lower levels of satisfaction in their personal relationships. They were also less likely to maintain these relationships, as highlighted by Guo et al.^38^ Furthermore, numerous studies have consistently demonstrated that individuals with robust social support networks possess a greater capacity to effectively manage the stressors that can trigger or worsen depressive symptoms McDonald et al.^39^ Moreover, those with ample social support are more likely to receive therapeutic interventions, which play a crucial role in alleviating and effectively managing their symptoms.

#### Stigma

Participants could not tell people they were depressed because they feared the stigma associated with mental illness. Furthermore, they considered living with depression to be a dishonour. This may be observed in the following quotation:

‘Yes, many, many people have discriminated against me for taking psychiatric medication.’ (Participant 3, 42-year-old, Male)

Another participant said:

‘When we are in the queue, they say that is sick is sick is sick, and you see my reputation is going down. Yes, they are killing my confidence of taking the medication.’ (Participant 1, 26-year-old, Male)

Mental illnesses significantly influence individuals’ lives and the lives of family members and friends, especially given the persistent stigma associated with depression.^18^ The extent to which depression is stigmatised by the public and the depth of internalised stigma for those living with depression has been a largely ignored topic.^40^ The impact of stigma on the lives and treatment results of individuals living with depression necessitates a concerted effort in mental health research and policy to address this issue.^41^

### Category 3: Social circumstances impacted adults living with depression’s compliance with medication

In this study, it became evident that a significant number of adults living with depression commonly experienced medication non-compliance because of the social circumstances they encountered. This non-compliance was often seen as a personal decision. Furthermore, negative experiences were found to increase the risk of depression. The adults living with depression shared their perspectives of their social circumstances which are discussed next.

#### A lack of support

In this study, most adults living with depression experienced poor social support, which raised their risk of recurrent depression and medication non-compliance, causing the buffering effect against stressors to decrease. A participant shared:

‘By not going there anymore. Nevertheless, I stopped going there. And then I think that made me leave this medication because people they are making me, they are making me a laughing stock at the community.’ (Participant 1, 26-year-old, Male)

Another participant reflected:

‘They did not treat me like they used to treat me, and I am not, I am not ill. I went there to get help.’ (Participant 2, 42-year-old, Female)

Research conducted on adults living with depression indicated that individuals who lack support from their family and friends experience a decline in their self-esteem, an increase in feelings of worthlessness, a heightened sense of guilt, elevated levels of stress, and an augmented likelihood of experiencing suicidal thoughts and engaging in self-destructive behaviours.^42^ Adults themselves have also reported that perceived social support plays a crucial role in effectively managing the symptoms associated with depression.^43^

#### Financial constraints

Adults living with depression mentioned being at risk of losing their jobs and receiving a lower income because of their mental illness, having to go for follow-up appointments and obtaining medication. Participants felt overwhelmed, and that affected their performance in the workplace. The following quote illustrates financial constraints and reduced income of adult participants with depression when dealing with their illness:

‘It has happened before where my finances have caused me not to be able to come to the facility to get medication. I leave the medication until I have enough money to be able to buy and get them because medication costs a lot of money.’ (Participant 5, 55-year-old, Female)

The majority of adults living with depression required time off from work, and one-third of adults living with depression had seen a pay drop or lost their employment since the onset of the condition.^44^ Participants were concerned about financial restrictions; they stated they had limited time to work to earn money because most of their time was spent travelling to the hospital, seeing physicians and acquiring medicine.^45^

## Theme 2: Adults living with major depression experienced non-compliance, which created a setback to their recovery

This study revealed that medication compliance is based on individual perceptions of depression and its treatment, and actual experiences with antidepressant treatment regimens. It was also observed that adults who receive support from their friends, family and community are more likely to realise the importance of continuing their medication. Moreover, those who experienced concern over their mental health were also more likely to comply with their medication. These are described below.

### Category 1: Perceptions of medication changed when adults living with depression realised that non-compliance impacted their lives

Dealing with mental health treatment services is complex, especially in determining effective treatment techniques.^46^ Most adults living with depression in this study changed their perception of their medication and believed that their treatment helped, and most received a combination treatment for depression. However, this study also discovered that low perceived effectiveness of treatment was associated with poor self-rated health and a likelihood of having severe mental illness and substance use problems. These issues are discussed next.

#### Experienced support from friends and family

Adults living with depression reported an unawareness of the signs and symptoms of depression, believing that their feelings were normal. Participants in this study acknowledged that support is important for them to continue taking their medication, and they realised that their depression symptoms may worsen without medication and therapy. One of the participants said:

‘To me, they support me completely. I just spoke to Nana, and she said I must get through. The community is very supportive. They want me to get to well. Yeah, they want me to continue taking your medication in a way they want you to be healthy here.’ (Participant 2, 42-year-old, Female)

Another participant reflected:

‘The realization that there are other people that matter is the one that can make me take my medication. My friends will help me see a doctor.’ (Participant 5, 55-year-old, Female)

From the foregoing excerpts, it is clear that participants appreciated receiving support and they also highlighted the need for continued support from family and friends. Manczak, Skerrett, Gabriel, Ryan and Langenecker^47^ argue that individuals who experience more conflict in the family and live in a less supportive environment are more likely to experience depressive symptoms; conversely, a positive family environment seems to be less likely to provoke mental health challenges issues. A supportive family environment is crucial for adults living with depression. By fostering open communication, family members can create safe spaces for adults living with depression to express their emotions and concerns without judgement.^48^ Furthermore, a recent study conducted in 2020 similarly emphasised the pivotal role family and friends played in providing emotional and informational support to individuals grappling with depression.^49^ This study highlighted how these close relationships served as invaluable sources of guidance and encouragement, aiding patients in making informed decisions regarding their choice of antidepressant medications, adhering to prescribed treatment plans and remaining committed to their medication regimen.^49^

#### Experienced concern over their mental health

Adults living with depression experienced concern over their mental health when they realised how it impacted their lives. They acknowledged that it is important to continue with their medication. One of the participants explained:

‘If you do take no medication normally, I am a chronic patient. I know that will end up relapsing and not maybe collapsing.’ (Participant 3, 42-year-old, Male)

Another added:

‘Because I know I needed medication to go on well, I cannot function 100% without falling into depression.’ (Participant 7, 48-year-old, Male)

The participants realised non-compliance to medication might have serious consequences, such as relapse. Compliance to medication is of utmost importance in ensuring the ongoing well-being and stability of adults living with depression. The antidepressants prescribed by healthcare professionals regulate neurotransmitter imbalances and alleviate the symptoms associated with various conditions, such as depression and anxiety.^50^ Moreover, by adhering to prescribed medication regimens, adults living with depression can experience improved cognitive functioning, stabilised mood, reduced stress levels, enhanced concentration and an overall better quality of life.^51^ Furthermore, compliance to medication helps prevent relapses and reduces the severity and frequency of symptomatology. Adults living with depression need to recognise that discontinuing or altering their medication without professional guidance can potentially result in various adverse effects, including withdrawal symptoms and a resurgence or intensification of mental health symptoms.

## Discussion

### Strengths and limitations

The recommendations provided for future changes are considered strengths of the study. A potential limitation was that the study was conducted in a single Gauteng mental health facility; hence, the findings are not representative of other Gauteng public hospitals’ psychiatric wards and cannot be generalised.

### Recommendations

The researcher suggests individualised patient-centred care should be emphasised for adults living with depression who are non-compliant with their psychiatric medication, as it promotes a therapeutic relationship and compliance with medication. The researcher further recommends development of an in-service training programme on medication compliance for all healthcare practitioners and that they should be encouraged to participate in these programmes actively. This will enhance healthcare practitioners’ expertise and will contribute to psycho-education for adults living with depression on compliance with psychiatric medication.

## Conclusion

This study aimed to explore and describe the experiences of adults living with depression who are non-compliant with their psychiatric medication and to formulate recommendations to facilitate compliance with their prescribed psychiatric medication. The results indicated that most negative experiences were associated with non-compliance with prescribed medications. The negative experiences of adults living with depression who are non-compliant with their psychiatric medication were also highlighted. This will ensure that resources are mobilised to promote and maintain medication compliance, and improve quality nursing care.
